# CHANGES IN RESIDENT OVARIAN MACROPHAGES DURING FEMALE REPRODUCTIVE AGING

**DOI:** 10.1093/geroni/igae098.2009

**Published:** 2024-12-31

**Authors:** Sarah Ocanas, Ewa Poljanska, Jose Isola, Chase Hubbart, Michael Stout

**Affiliations:** Oklahoma Medical Research Foundation, Oklahoma City, Oklahoma, United States; Oklahoma Medical Research Foundation, Oklahoma City, Oklahoma, United States; Oklahoma Medical Research Foundation, Oklahoma City, Oklahoma, United States; Oklahoma Medical Research Foundation, Oklahoma City, Oklahoma, United States; Oklahoma Medical Research Foundation, Oklahoma City, Oklahoma, United States

## Abstract

Ovaries exhibit accelerated aging phenotypes, displaying aging hallmarks earlier than other organs. During pre-menopause, endocrine function declines, and the ovarian microenvironment becomes pro-inflammatory and fibrotic. However, the cellular mechanisms driving ovarian aging remain unclear, hindering the development of therapies to delay this process for overall health benefits. Macrophages are crucial in ovarian functions like folliculogenesis, ovulation, and corpus luteum formation. Ovarian macrophages have two origins: tissue-resident macrophages (TRMs) from yolk sac and fetal liver progenitors, and monocyte-derived macrophages (MDMs) from bone marrow. In this study, we examined the transcriptomic profiles of ovarian TRMs in young (5-6 months) and older (12-14 months) Cx3cr1-NuTRAP mice. This mouse model uses a tamoxifen-inducible, cre-lox system to label nuclei and ribosomes, enabling DNA/RNA isolation without cell sorting. Flow cytometry confirmed the identity of eGFP-labeled cells, and translating ribosome affinity purification (TRAP) was used to assess age-related transcriptomic changes in TRMs. We found that TRMs exhibit inflammatory (e.g., IL1B) and senescent (e.g., CDKN2A) transcriptomic signatures, likely contributing to aging hallmarks such as T cell accumulation, multinucleated giant cell formation, and fibrosis. Translatability was established by comparing our data with publicly available human data. These findings suggest that ovarian TRMs play a critical role in ovarian aging, warranting future mechanistic studies.

